# Thyroid hormones induce doxorubicin chemosensitivity through enzymes involved in chemotherapy metabolism in lymphoma T cells

**DOI:** 10.18632/oncotarget.26890

**Published:** 2019-04-30

**Authors:** María Celeste Díaz Flaqué, Maria Florencia Cayrol, Helena Andrea Sterle, María del Rosario Aschero, Johanna Abigail Díaz Albuja, Blanca Isse, Ricardo Norberto Farías, Leandro Cerchietti, Cinthia Rosemblit, Graciela Alicia Cremaschi

**Affiliations:** ^1^ Instituto de Investigaciones Biomédicas (BIOMED), Consejo Nacional de Investigaciones Científicas y Técnicas (CONICET), Facultad de Ciencias Médicas, Pontificia Universidad Católica Argentina (UCA), Buenos Aires, Argentina; ^2^ Departmento de Bioquimica Nutricional, CONICET, Universidad Nacional de Tucuman, Instituto de Quimica Biologica “Dr Bernabe Bloj”, San Miguel de Tucuman, Tucuman, Argentina; ^3^ Division of Hematology and Oncology, Department of Medicine, Weill Cornell Medical College, Cornell University, New York, NY, USA

**Keywords:** thyroid hormones, CYP 450, integrin αvβ3, doxorubicin, T-cell lymphoma

## Abstract

Thyroid hormones (THs) – 3,3′,5-triiodo-L-thyronine (T3) and L-thyroxine (T4) – are important regulators of the metabolism and physiology of most normal tissues. Cytochrome P450 family 3A members are drug metabolizing enzymes involved in the activation and detoxification of several drugs. CYP3A4 is the major enzyme involved in the metabolism of chemotherapeutic drugs. In this work, we demonstrate that THs induce a significant increase in CYP3A4 mRNA levels, protein expression and metabolic activity through the membrane receptor integrin αvβ3 and the activation of signalling pathways through Stat1 and NF-κB. We reasoned that TH-induced CYP3A4 modulation may act as an important regulator in the metabolism of doxorubicin (Doxo). Experiments *in vitro* demonstrated that in CYP3A4-knocked down cells, no TH-mediated chemosensitivity to Doxo was observed. We also found that THs modulate these functions by activating the membrane receptor integrin αvβ3. In addition, we showed that the thyroid status can modulate CYP450 mRNA levels in tumor and liver tissues, and the tumor volume in response to chemotherapy *in vivo*. In fact, Doxo treatment in hypothyroid mice was associated with lower tumors, displaying lower levels of CYP enzymes, than euthyroid mice. However, higher mRNA levels of CYP enzymes were found in livers from Doxo treated hypothyroid mice respect to control. These results present a new mechanism by which TH could modulate chemotherapy response. These findings highlight the importance of evaluating thyroid status in patients during application of T-cell lymphoma therapeutic regimens.

## INTRODUCTION

T-cell lymphomas (TCL) are a heterogeneous group of aggressive lymphoproliferative disorders with considerable clinical, morphological, immunophenotypic and genetic variation, including approximately 10–15% of all lymphoid neoplasms [1]. Despite producing less than satisfactory results, the combination of cyclophosphamide, doxorubicin, vincristine and prednisone (CHOP) remains the standard treatment for TCL [2]. This therapeutic approach, originally designed for B-cell lymphomas, has poor outcome with common recurrence and few effective options for rescue therapy [2, 3]. This fact clearly points out the importance of identifying therapies to improve disease outcome. Cytochrome P450 family 3A members (CYP3A4, 5, 7 and 43) are drug metabolizing enzymes involved in the activation and detoxification of several drugs [4]. Due to its ability to activate or inactivate chemotherapeutic drugs, the expression of CYP has been proposed as a biomarker of the clinical evolution in patients treated with chemotherapy [5]. Moreover, CYP enzymes have been shown to either activate some anticancer drugs, or to inactivate other anticancer agents [6]. Although the regulation of CYP enzymes exerted by hormonal nuclear receptors has been well studied [7], few studies have explored the regulation of these genes by thyroid hormones and their receptors in pathological conditions.

Thyroid hormones (THs), 3,5,3′-triiodo-L-thyronine (T3) and L-thyroxine (T4), are essential regulators of the differentiation, growth, metabolism and physiological functions of most normal tissues. In our previous work we have shown that THs have been implicated in cell transformation, tumorigenesis, angiogenesis [8]; and metastatic spread [9]. Particularly, it has been shown that THs action on these biological processes can be mediated through its nuclear TRα receptor, but also via nongenomic mechanisms, involving cell surface membrane receptors (mTR) such as integrin αvβ3 and cytoplasmic located TR [10–14]. *In vitro* studies carried out in our laboratory have shown that THs, via a membrane receptor, could modulate the balance between proliferation and viability versus apoptosis of TCL cells involving different intracellular signaling pathways like p42/44 MAPKs, PKC, and NF-kB [8, 15–17]. In this sense, other authors have shown a nuclear association between integrin αv, phosphorylated p42/44 MAPKs and Stat1 in T4-stimulated OVCAR-3 cells [18].

There are some studies describing genetic polymorphisms of CYP enzymes in patients with Non-Hodgkin’s lymphomas [19], as well as the association between side effects of drugs for leukemia treatment and the expression of these enzymes [20]. However, studies evaluating the regulation of CYP enzymes by THs and the response to the therapy were not performed yet.

In this study, we analyzed the effect of THs on therapy response and the modulation of enzymes involved in the metabolism of chemotherapeutic drugs. We here show, for the first time, that THs can induce CYP3A4 expression in TCL cells, having different effects on CHOP drugs depending on whether their metabolites are active or inactive, Furthermore, these effects were confirmed *in vivo* and the relevance of the patient thyroid status in chemotherapy outcome was discussed here as well.

## RESULTS

### THs induce chemo-sensitivity to doxorubicin in TCL cells

**Figure 1:**
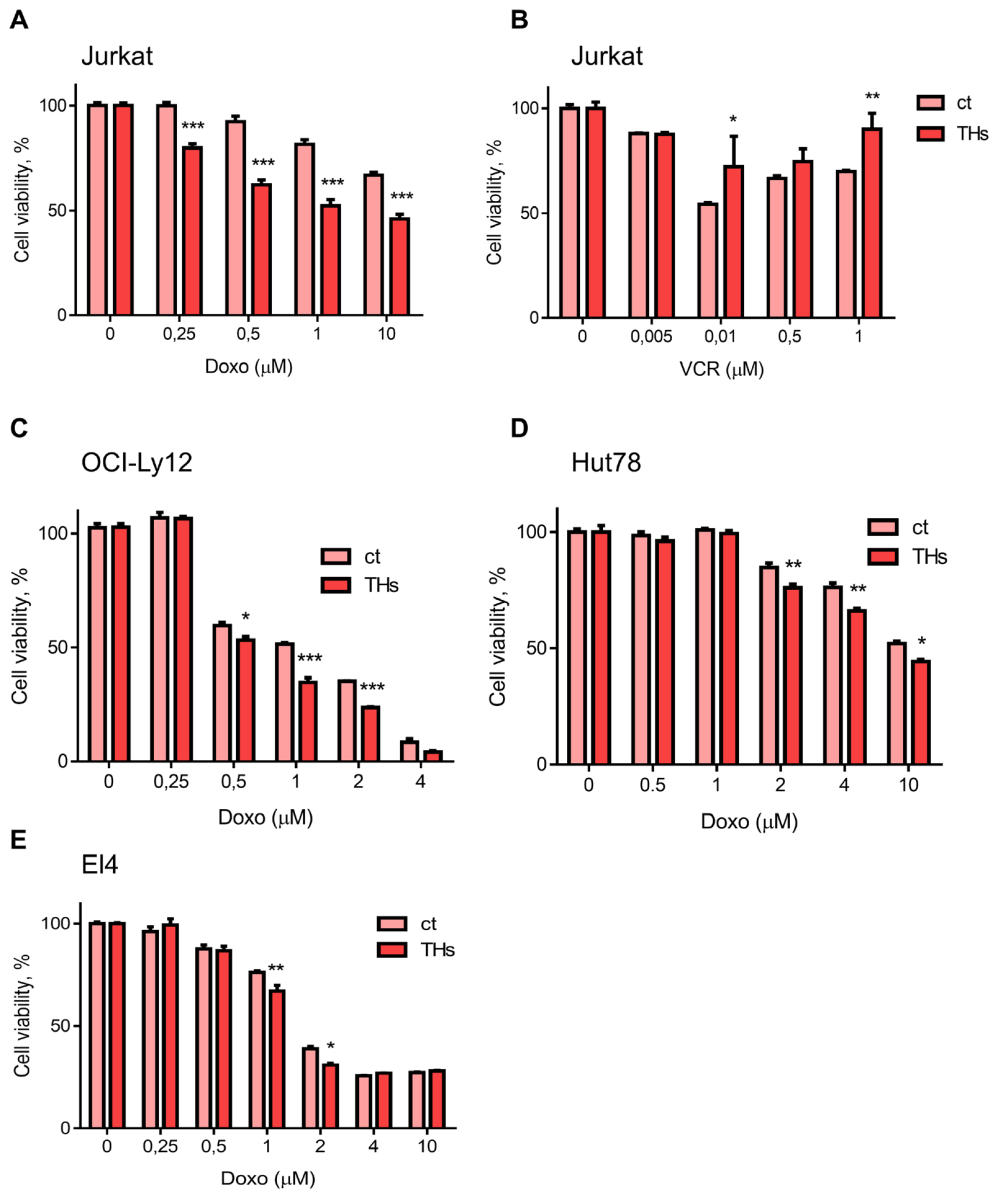
Thyroid hormones sensitize T lymphoma cells to doxorubicin treatment. Jurkat cells were pretreated or not (ct) with THs for 18 h before treatment with different doses of Doxo (**A**) or VCR (**B**). OCI-Ly12 (**C**), Hut78 (**D**) and El4 (**E**) cells were pretreated or not (ct) with THs for 18 h before treatment with different doses of Doxo. Cell Titer Blue assay determined the number of live cells at each dose. Data are shown as mean ± SD. ^*^p < 0.05; ^**^p < 0.01; ^***^p < 0.001 respect to untreated cells.

To assess the effect of THs on the response to conventional chemotherapy, we treated Jurkat cells with increasing doses of doxorubicin (Doxo) and vincristine (VCR) in presence or absence of physiological concentrations of T3 and T4 (1 nM and 100 nM, respectively) to mimic circulating levels of both hormones. We used these two chemotherapeutic drugs as Doxo render an active metabolite, while VCR metabolite is inactive [21]. As expected, Doxo and VCR induce cytotoxicity in TCL cells treated or not with THs (Figure 1A and 1B). Doxo significantly decreased cell viability in a dose-dependent manner, and this effect was even greater in presence of THs (Figure 1A). On the other hand, VCR effects on cell viability were reverted when Jurkart cells were pretreated with THs (Figure 1B). Similar results were found in Doxo-treated OCY-Ly12 cells (Figure 1C), Hut78 (Figure 1D) and in the murine lymphoma T cell line EL4 (Figure 1E).

**Figure 2:**
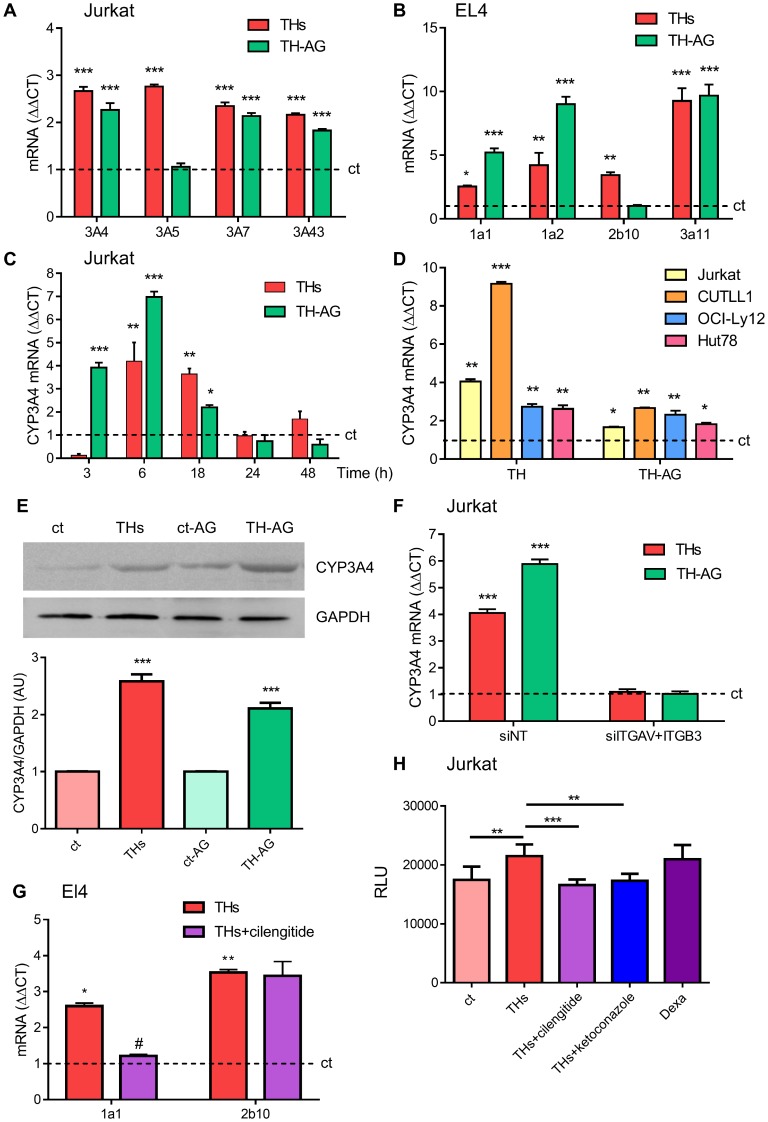
Thyroid hormones modulation of CYP P450 levels. Role of the integrin αvβ3 TH membrane receptor. (**A**) mRNA levels of CYP3A4, CYP3A5, CYP3A7 and CYP3A43 were analyzed by qPCR in Jurkat cells treated with THs or TH-AG for 18 hs. (**B**) mRNA levels of CYP1a1, CYP1a2, CYP2b10 and CYP3a11 were analyzed by qPCR in EL4 cells treated with THs or TH-AG for 18 hs. (**C**) mRNA levels of CYP3A4 were analyzed by qPCR in Jurkat cells treated with THs or TH-AG for the indicated times. (**D**) mRNA levels of CYP3A4 were analyzed by qPCR, in Jurkat, CUTTL1, OCI-Ly12 and Hut78 cells treated with THs or TH-AG for 18 hs. (**E**) Jurkat cells were treated with THs, TH-AG, AG (ct-AG) or remained untreated (ct) for 24 h. Western Blot analysis shows CYP3A4 levels. GAPDH was used as loading control. Top panel: representative data from 1 of 3 independent experiments. Bottom panel: densitometric analysis. (**F**) Jurkat cells transfected with non-target siRNA (siNT, control), anti-integrin αv + anti-integrin β3 siRNA (siITGAV+ITGB3) were treated with THs, TH-AG or remained untreated for 18 h. mRNA levels of CYP3A4 were analyzed by qPCR. (**G**) mRNA levels of CYP1a1 and CYP2b10 were analyzed by qPCR in EL4 cells treated with THs, THs + 1,5 mM cilengitide. (**H**) CYP3A4 enzymatic activity was measured using P450-Glo kit Assays in Jurkat cells that were treated or not with THs, THs + 1,5 μM cilengitide, THs + 1 μM ketoconazole or 50 μM Dexametasone for 48 h. Data are shown as mean ± SD. ^*^p < 0.05; ^**^p < 0.01; ^***^p < 0.001 vs. untreated cells (ct, dash line), *^#^p < 0.01* vs. THs treated cells.

Considering the results shown above, we investigated the metabolizing enzymes of chemotherapeutic drugs. Thus, we evaluated THs regulation of CYP3A family members (CYP3A4, CYP3A5, CYP3A7, and CYP3A43). We found that every CYP3A gene was significantly modulated by THs in Jurkat cells treated for 18 h (Figure 2A). In mice, CYP1A1, CYP1A2, CYP2b10 and CYP3A11 isozymes have been described as responsible for the metabolism of xenobiotic compounds [22]. The mRNA levels of the four enzymes were up-regulated *in vitro* by THs in EL4 cells (Figure 2B).

Since CYP3A4 metabolizes more than 50% of drugs currently used in chemotherapy [4] and its expression in peripheral T-cell lymphoma (PTCL) is associated with a shorter survival rate [5], we focused our experiments on this member of the CYP3A family. In this line, to elucidate the participation of THs classic and nongenomic effects we performed *in vitro* assays with THs or THs-coupled to agarose (TH-AG), the latter unable to pass through the plasma membrane and thus binding exclusively to the mTR, the integrin αvβ3 [17]. Treatment with both THs and TH-AG for 6 h-induced a significant increase on CYP3A4 mRNA synthesis (Figure 2C). Similar results were found in CUTLL1, OCI-Ly12 and Hut78 cell lines (Figure 2D). In addition, 24 h treatment with physiological concentrations of THs and TH-AG increased protein levels of CYP3A4 by western blot assays in Jurkat cells (Figure 2E). These results are in line with those obtained for CYP3A4 mRNA levels, suggesting that THs induce modulation at both mRNA and protein CYP3A4 levels.

We have previously demonstrated the functionality of integrin αvβ3 as membrane receptor for TH-induced angiogenesis and proliferation in TCL cells [8]. To confirm that integrin αvβ3 is critical on TH-induced CYP3A4 expression, we performed experiments inhibiting expression of this integrin. For this purpose, Jurkat cells were transfected with si-ITGAV+si-ITGB3. Down-regulation of the integrin αvβ3 inhibited both THs and TH-AG-induced CYP3A4 mRNA expression (Figure 2F). Similar results were found in EL4 cells treated with cilengitide, a cyclic RGD pentapeptide that inhibit αvβ3 integrin [23] (Figure 2G).

We then analyzed whether TH-dependent increase on CYP3A4 protein levels could modulate its enzymatic activity assayed by P450-Glo kit assay. As shown in Figure 2H, THs induced metabolic activation of CYP3A4 respect to untreated cells reaching similar levels to those induced by dexamethasone, a well-known CYP3A4 inducer [24]. Also, CYP3A4 inhibitor ketoconazole [25] prevented TH-induced CYP3A4 activation. In the presence of cilengitide, THs were not able to induce CYP3A4 enzymatic activity (Figure 2H).

### THs and agarose-coupled THs induce signaling pathway activation in TCL cells

**Figure 3:**
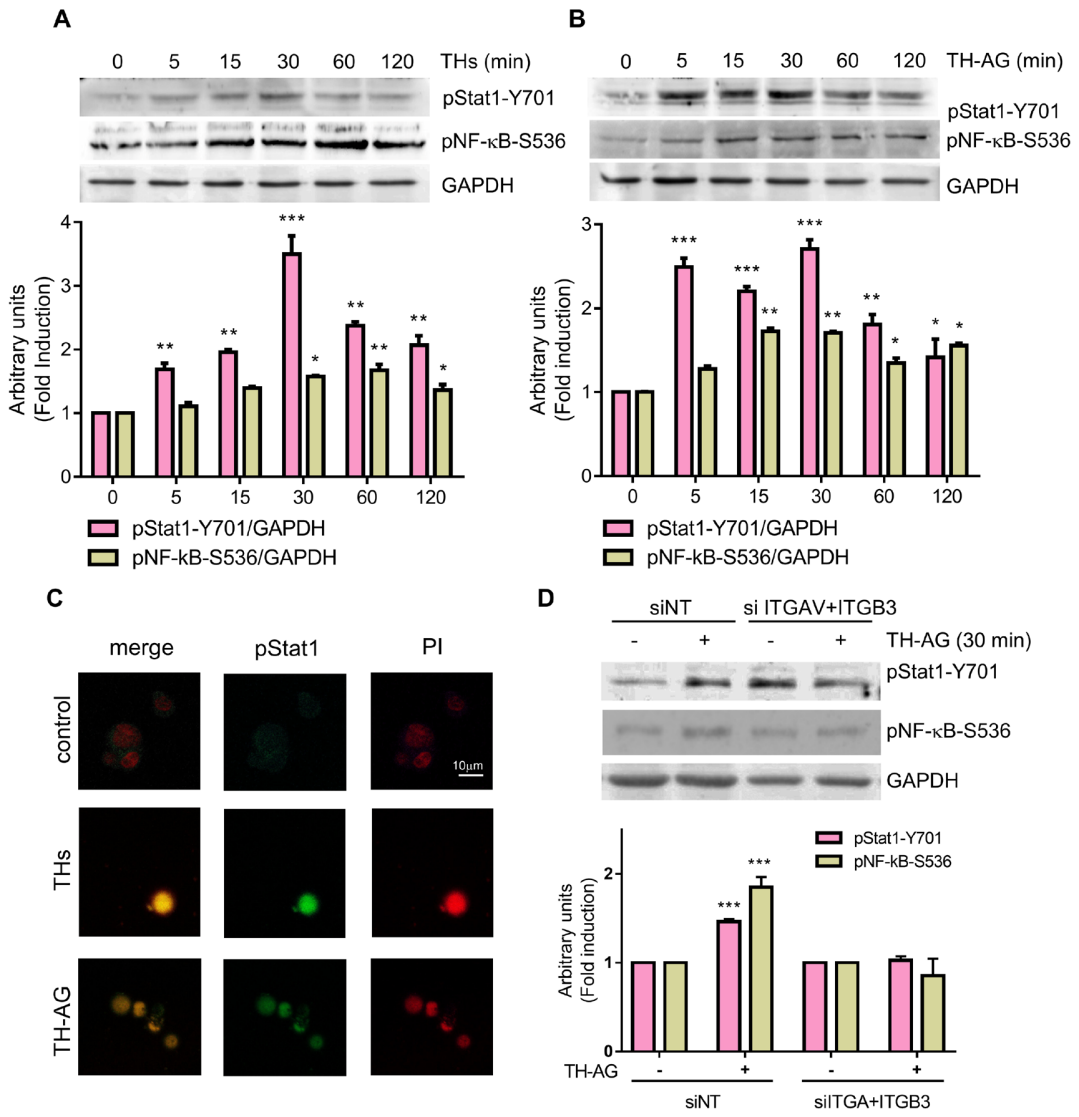
Thyroid hormones induce Stat1 and NF-kB activation. Jurkat cells were treated with THs (**A**), TH-AG (**B**) or remained untreated (ct) for the indicated times. Western Blot analysis show Stat1-Y701-phosphorylation and NF-kB-S536-phosphorylation levels. GAPDH was used as loading control. Top panel: representative data from 1 of 3 independent experiments. Bottom panel: densitometric analysis. (**C**) Representative photograph of pStat1 nuclear localization as analyzed by IF and confocal microscope an cells treated with THs, TH-AG, or untreated (control) for 15 min. (**D**) Cells transfected with non-target siRNA (siNT) or anti-integrin αv + anti-integrin β3 siRNA (siITGAV+ITGB3) were treated with TH-AG or remained untreated for 30 min. Western Blot analysis show Stat1-Y701-phosphorylation and NF-kB-S536-phosphorylation levels. GAPDH was used as loading control. Top panel: representative data from 1 of 3 independent experiments. Bottom panel: densitometric analysis. Data are shown as mean ± SD. ^**^p < 0.01; ^***^p < 0.001 vs. untreated cells.

Our group has previously demonstrated that THs and TH-AG induce p42/44 MAPK phosphorylation and NF-κB activation down-stream PKCζ in murine T lymphoma cells [15]. In addition, we have previously demonstrated that THs induce NF-κB activation and nuclear translocation in human TCL [8]. As shown in Figure 3A and 3B, THs and TH-AG significantly increased NF-κB phosphorylation in Ser 536. Previous work showed that THs induce Stat1 phosphorylation [26]. In this sense, here we show that THs and TH-AG induced Stat1 phosphorylation in Tyr 701, as well (Figure 3A and 3B). Both THs and TH-AG induced Stat1 nuclear translocation (Figure 3C).

**Figure 4:**
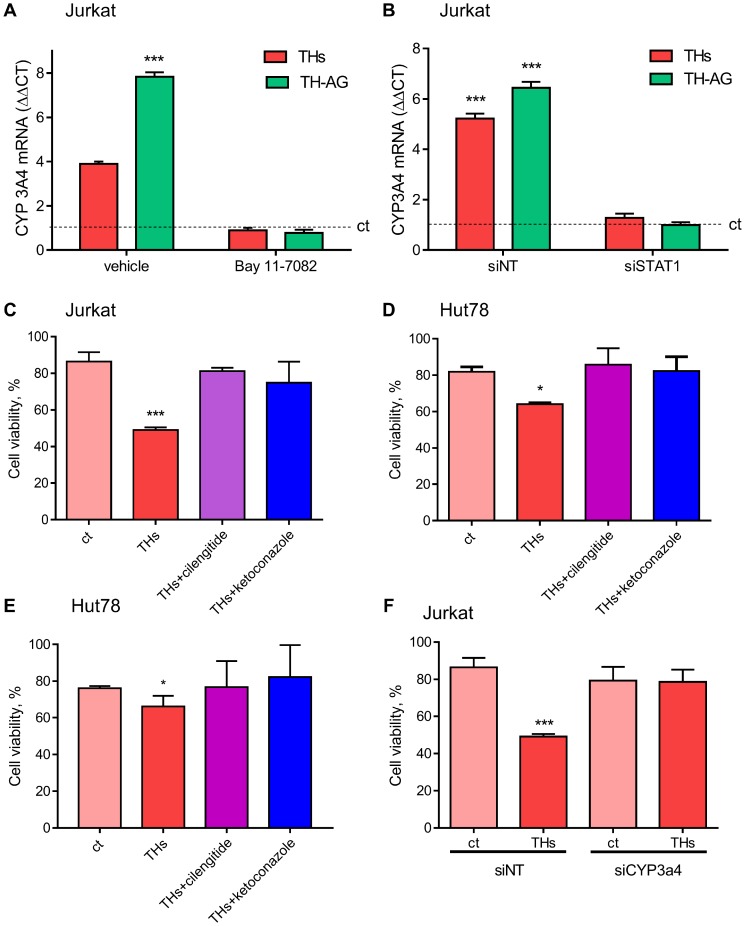
Thyroid hormones modulate CYP3A4 expression and TH-induced doxorubicin chemo-sensitivity. (**A**) Cells were pre-treated with vehicle or BAY 11-7082 (2.5 μM) and then treated for 18 with THs, TH-AG, or remained untreated (control, dash line). We analyzed mRNA levels of CYP3A4 on these conditions. (**B**) mRNA levels were measured on cells previously transfected with non-target siRNA (siNT) or anti Stat1 siRNA (siStat1) and then incubated with THs or TH-AG as indicated above. Data are shown as mean ± SD. ^***^p < 0.001 respect to untreated cells. Cell Titer Blue assay was performed in Jurkat (**C**), HuT78 (**D**) and El4 (**E**) cells pre-treated with THs, TH + cilengitide 1.5 μM or THs + ketoconazole 1 μM for 6 h or remained untreated (ct) and then treated with Doxo 1 μM for 24 h, and Jurkat cells (**F**) transfected with siNT or anti CYP3A4 siRNA (siCYP3A4) were pre-treated or not (ct) with THs for 6 h and then treated with Doxo 1 μM for 24 h. Data are shown as mean ± SD. ^***^p < 0.001 respect to ct.

Giving that TH-AG are not able to enter the cell, these results suggest the activation of a membrane receptor that induces intracellular signaling pathways. To confirm that integrin αvβ3 is the membrane receptor involved in TH-induced signaling pathway, we evaluated the effect of THs in integrin αvβ3-depleted cells. We found that 30 min of TH-AG were not able to induce neither Stat1 nor NF-κB (Figure 3D). As expected, the NF-κB inhibitor (BAY-11-7082) and treatment with si-Stat1 impairs both THs and TH-AG-induced expression of CYP3A4 mRNA (Figure 4A and 4B).

### TH-mediated Doxo chemosensitivity occurs through integrin αvβ3 activations and CYP modulation

To corroborate the biological significance of TH-induced CYP3A4 expression through the integrin αvβ3 in Doxo sensitization, we analyzed cell viability. As shown in Figure 4C, in presence of integrin αvβ3 inhibitior cilengitide, THs did not sensitize Jurkat cells to Doxo, reaching the same level as ketoconazole, a pharmacological inhibitor of CYP3A4. Similar results were found in Hut78 and El4 (Figure 4D and 4E). In the same way, inhibiting the expression of CYP3A4 with siRNA, THs were unable to sensitize Jurkat cells to Doxo treatment (Figure 4F). These results suggest that THs modulate the CYP3A enzyme through its non-classical membrane receptor affecting the efficacy of Doxo.

### Thyroid status impact on cytochrome P450 expression *in vivo* and affect therapy response

**Figure 5:**
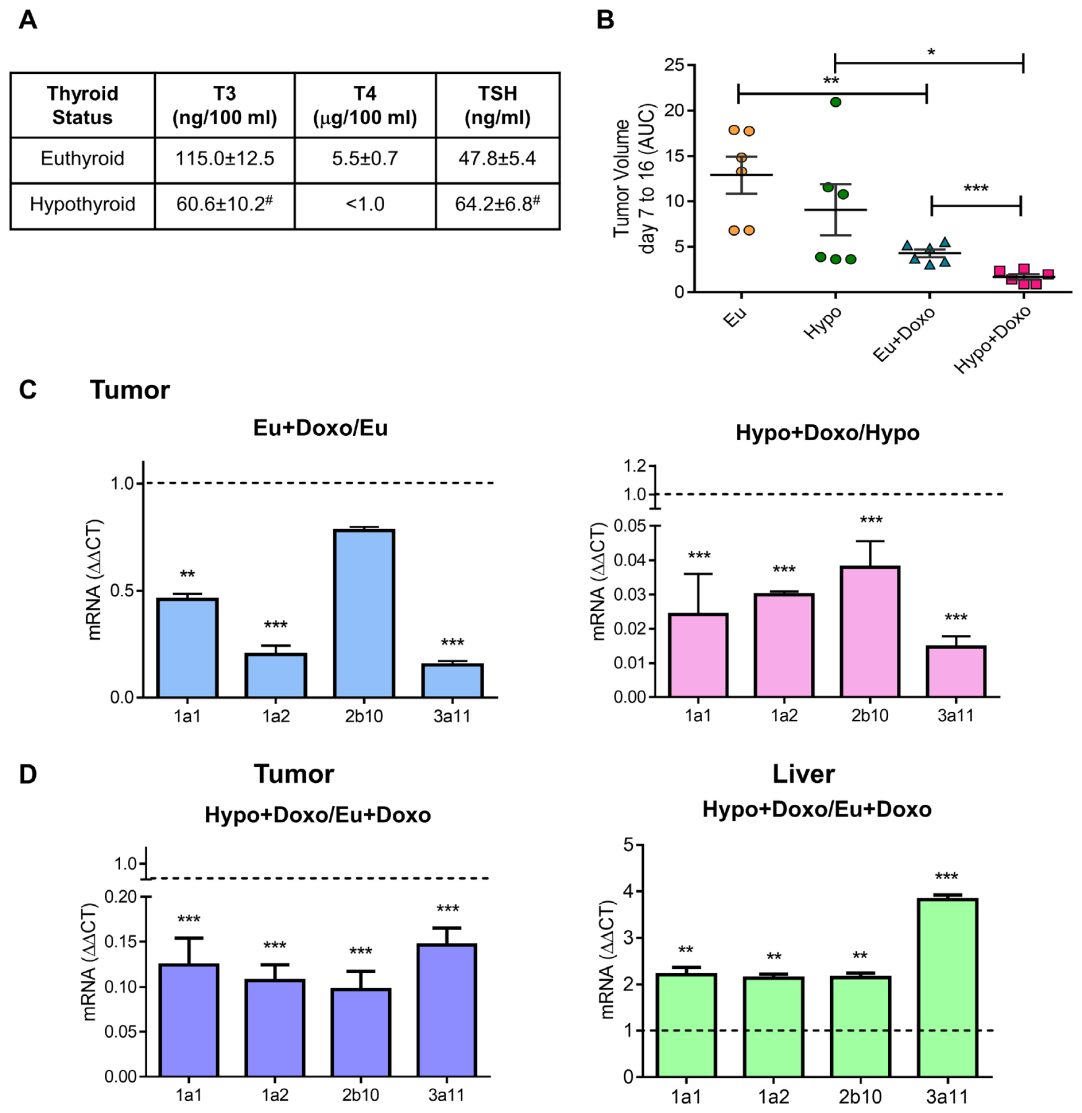
Effect of thyroid status on murine cytochrome P450 expression and tumor response to chemotherapy. (**A**) Plasma levels of THs in Euthyroid, Hyperthyroid, and Hypothyroid mice were determined using ELISA. #*p* < 0.05 (**B**) Euthyroid, and hypothyroid mice were subcutaneously inoculated with 3 × 10^5^ EL4 cells (*n* = 5 for each group). When tumors were palpable, mice were randomized and treated or not with Doxo. Tumors growth from day 7 to 16 post inoculation was measured by the AUC. Data are shown as mean ± S.E.M ^*^p < 0.05 respect to Hypothyroid animals, ^**^p < 0.01 respect to euthyroid animals or ^***^p < 0.001 respect to Euthyroid+Doxo animals. mRNA expression of murine CYP P450 1A1, 1A2, 2b10 and 3A11 was determined in tumor and liver tissues (**C** and **D**) by qPCR analysis. Gene expression was normalized to β-2-microglobulin gene, and the ΔΔCt method was used to calculate the fold change. Data are shown as mean ± S.E.M ^**^p < 0.01 or ^***^p < 0.001.

In previous sections, we described how THs induce CYP3 *in vitro* regulation in human and murine TCL cells and its importance in Doxo metabolism. To address whether circulating levels of THs modulate CYP expression and Doxo response, we evaluate tumor growth in tumor-bearing euthyroid (Eu) and hypothyroid (Hypo) mice. EL4 lymphoma cells were inoculated in syngeneic mice with different thyroid status as previously described [9]. T3, T4, and TSH plasma levels were determined to check the efficacy of T4 and PTU treatments. As previously reported by our group [9], hypothyroid mice showed lower T3 and T4 levels, but higher levels of TSH than euthyroid mice (Figure 5A). After EL4 cell inoculation all animals developed solid tumors. When the tumors reached a palpable size, mice of each thyroid status were randomly separated in two groups: one remains untreated (Eu and Hypo) and the others were treated with Doxo (Eu+Doxo and Hypo+Doxo). At day 14 post injection (p.i.), Eu+Doxo mice showed a significant decrease in EL4 lymphoma growth compared with tumors in control euthyroid mice (Figure 5B) indicating the effectiveness of Doxo treatment. As we had previously reported, Hypo mice did not show significant differences in tumor volume respect to the Eu mice [9], but Doxo was also effective in these mice as well. However, unlike what we expected according to our *in vitro* results of viability, the tumor volume in Hypo+Doxo mice was significantly lower than Eu+Doxo mice (Figure 5A).

To elucidate whether THs circulating levels modulate Doxo-response, mRNA from tumors was extracted from mice with different thyroid status and CYP expression was analyzed. As seen in Figure 5C, Doxo decreased mRNA levels of the analyzed isozymes both in euthyroid and hypothyroid conditions. However, Doxo-mediated decrease in Hypo mice was significantly lower than Eu+Doxo mice (Figure 5D). As *in vivo* CYP metabolism, is mainly related to the higher protein content of CYP in adult liver [4], we evaluated hepatic CYP mRNA levels in Doxo-treated mice with normal or low levels of THs. In the liver, a significant increase in the four CYP enzymes was found under hypothyroid conditions respect to euthyroidism (Figure 5D). So, higher hepatic metabolism of CYP in Hypo-Doxo mice, rendering high circulating levels of the active metabolite, would account for the low tumor volumes found in this condition, thus pointing out the importance of thyroid status on chemotherapy outcome.

## DISCUSSION

The knowledge of the molecular mechanisms leading to tumor chemotherapy resistance is crucial to assure the success of treatment. Among these mechanisms, factors that influence CYP expression and function play an important role. In this work, we demonstrate the regulatory role of THs, at the physiological concentrations found in tumor microenvironment, on response to Doxo in TCL cells. This effect is mediated by Stat1 and NF-κB activation leading to CYP3A4 expression and to the up-regulation of its enzymatic activity. The viability studies revealed that under proliferative conditions pretreatment with THs sensitizes Jurkat cells to Doxo treatment, but not to VCR. This could be related to the fact that Doxo metabolites are active while this is not the case with VCR [21].

The cytochrome P450 is a large and diverse superfamily of hemoproteins using a wide range of exogenous and endogenous compounds as substrates for their enzymatic reactions. The CYP3A subfamily, consisting of the isozymes CYP3A4, CYP3A5, CYP3A7 and CYP3A43 is responsible for the metabolism of approximately 50% of the drugs currently used [4]. All members of CYP3A family were found up-regulated by THs in TCL cells. Being CYP3A4 the major enzyme involved in the metabolism of chemotherapeutic drugs, we reasoned that TH-induced CYP3A4 metabolic activity may act as an important regulator in the metabolism of Doxo in our experimental conditions. Here we demonstrate that THs induce CYP3A4 expression through the activation of non-classical THs receptors, namely the integrin αvβ3 as demonstrated by the genomic down-regulation of the mTR. In concordance, our previous work demonstrates that THs exert non-classical actions through the integrin αvβ3 in several TCL cell lines [8]. THs are present in the cell microenvironment and can exert nongenomic actions in a short period of time [11]. The nongenomic mechanism of action of THs are generally mediated by rapid signaling pathways including the activation of PKC, PKA, PI3K, protein kinase, MAPK and the activation of PLC, among others [11, 12]. Additionally, THs may alter intracellular trafficking and the phosphorylation of TR, ERα, Stat1 and p53, through the integrin αvβ3 pathway [27] and nuclear interaction of Stat1 with the integrin αv monomer in ovarian cancer cells [18]. Previous work from our laboratory shows that THs can stimulate the proliferation of murine TCL via THs binding to its membrane receptor and activating NF-κB as a transcription factor [17]. Furthermore, it was shown that the THs acting at physiological concentrations can induce angiogenesis and proliferation of human mature and immature TCL. In summary, our results indicate that THs acting through its membrane receptor integrin αvβ3 can activate the transcription factors Stat1 and NF-κB leading to the increase CYP3A4 expression levels downstream.

We found that cilengitide impairs THs sensitization of TCL cells to Doxo treatment. Cilengitide, is a small cyclic peptide ligand, that recognized RGD extracellular domain of integrin αvβ3, currently used in clinical studies [23]. Promising results were found in advanced solid tumors treated in combination with paclitaxel [28] and chemoradiotherapy [29]. These results are in concordance with the fact that THs are not able to induce CYP3A4 expression in the presence of integrin αvβ3 down-regulation.

**Figure 6:**
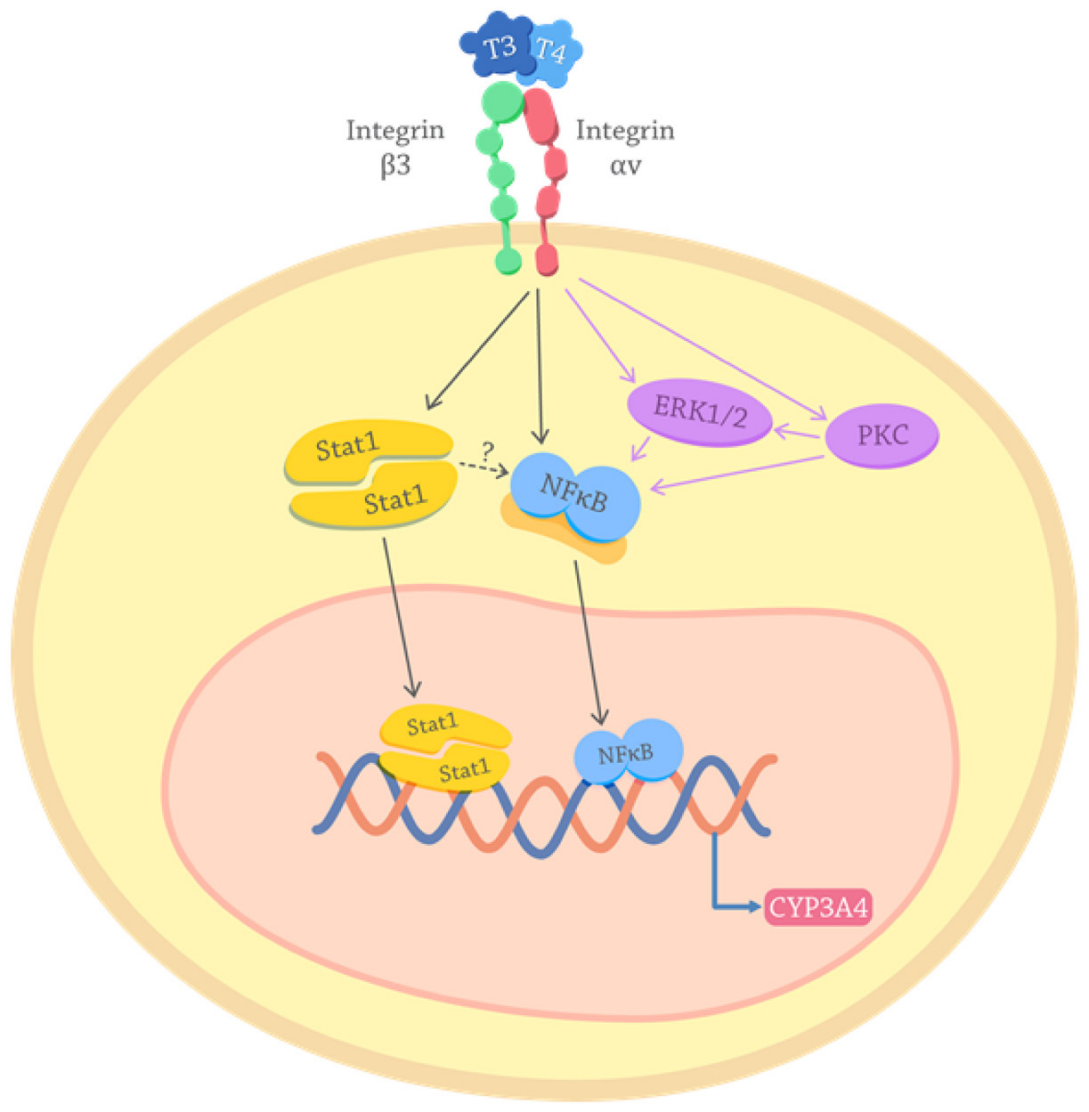
Proposed mechanisms for the effects of thyroid hormones on the CYP3A4 expression. THs interact with the receptor integrin αvβ3 on the cancer cell membrane and activate intracellular pathways. Purple arrows depict PKC and ERK pathways described previously in TCL cells [8, 17, 58]. Black arrows show the signals here elucidated: THs induction of Stat1 and NF-κB activation. Once Stat1 and NF-κB are activated translocate to the nucleus where they act as transcription factors that lead to the genomic transcription of CYP3A4. The possible activation of NF-kb downstream Stat1 is also suggested (dotted arrow).

The proposed molecular mechanism by which THs induce CYP3A4 modulation is depicted in Figure 6. T3 and T4 interacting with integrin αvβ3 in the membrane cell induce Stat1 and NF-κB phosphorylation and nuclear translocation leading to CYP3A4 expression. It is important to note, that in silico analyzes (Genomatix genome analyser -MatInspector. http://www.genomatix.de.) strengthen the fact that THs action on CYP is indirect via the participation of transcription factors activated through the membrane receptor, as these assays indicate an absence of THs response elements (TRE) in the CYP3A4 proximal promoter.

In this work, in addition to THs involvement in CYP3A4 expression and activation, we also demonstrate a THs role in response to chemotherapeutic drugs. Both Doxo and VCR are metabolized by enzymes of the CYP3A family, being CYP3A4 the primary isoforms that mediate their biotransformation [30]. In the case of Doxo, CYP3A4-mediated metabolism leads to drug activation, whereas VCR is inactivated [21]. Doxo is metabolized by liver CYP450 enzymes which release the activated metabolite into the systemic circulation with therapeutic activity, but also with associated host cell toxicity [31]. The effectiveness of Doxo in destroying cancer cells have been widely shown; however, due to off-target complications as cardiomyopathies, it should be use dose-limited [32]. CYP3A isoforms are mainly expressed in the liver [33], however, some specific isoforms are further expressed extrahepatically [34, 35]. The expression of different CYP3A genes was demonstrated in a subset of PTCL tumors [5], and also in various cancers, including breast, lung, colon and those of the head and neck, showing differential expression of CYP450 levels in tumor relative to normal tissue [36–39]. In this way, several authors have proposed the P450-Based Gene-Directed Enzyme Prodrug Therapy to potentially enhance the efficacy of P450-activated anticancer prodrugs, without increasing host toxicities [40–44]. In this work we showed that the thyroid status can modulate the *in vivo* response to Doxo and CYP levels both in tumor and liver tissue. In fact, tumor-bearing hypothyroid mice display a greater response to Doxo treatment with smaller tumors than euthyroid Doxo-treated mice. To this end, thyroid status regulation of CYP enzymes was evaluated showing that Doxo decreased CYP levels both in tumors of mice with euthyroidism and with hypothyroidism, being significantly lower in the latter. This would agree with the increase in CYP levels induced *in vitro* in EL4 cells by THs. Additionally, this would also be related to the tumor volume found in Hypo+Doxo mice, as a low biotransformation of the active metabolite is expected.

Other authors have reported hypothyroidism as a positive prognostic factor for the outcome of the disease in different types of cancer even when hypothyroidism occurs as a side effect of the treatment received [45, 46]. This could indicate that the lower tumor growth seen in Doxo-treated hypothyroid mice is due to the effect of the thyroid status by itself. However in our model we have not seen differences in the tumor size between hypothyroid and euthyroid mice [9].

We even found that Hypo+Doxo mice have higher levels of CYP enzymes in the liver than Eu+Doxo, which would also point to higher circulating levels of the active metabolite, thus contributing to the low tumor volume found in response to Doxo in hypothyroid mice. In support to our results it has been shown that Doxo decreases the hepatic expression of CYP enzymes in rats [47] and that THs can decrease CYP levels in human hepatocytes *in vitro* [48], so it would be expected that low circulating levels of them may increase hepatic CYP. Moreover, the effect of thyroid function on the efficacy of cancer therapies has been described and hypothyroidism was suggested to have a positive impact on treatment outcome [45, 49, 50]. Furthermore, several authors have demonstrated shown that CYP3A4 has a differential expression in normal tissue vs tumor samples. Thus, Trujillo-Paolillo *et al.* [51] have demonstrated that Osteosarcoma biopsy specimens has lower CYP3A4 levels than normal bone specimens. Similar findings have been found for CYP3A4 in Hepatocellular Carcinoma tissues as compared to paired adjacent noncancerous liver tissues [52, 53].

In summary, we found that TH-mediated non-classical actions could affect conventional chemotherapy outcomes. Together, these results highlight the importance of personalized medicine. The knowledge of the patient thyroid status and the possible modulation of drug metabolizing enzymes by thyroid hormones in the tumor could help to take a better choice of the chemotherapeutic regimen to each patient with the consequent success of the therapy.

## MATERIALS AND METHODS

### Preparation of T3 and T4 agarose-bound

Agarose-bound triiodothyronine and thyroxine hormones (T3-AG and T4-AG, respectively) were obtained as previously described [14]. Briefly, T3 and T4 were purchased from Sigma Chemical Co. (St. Louis, MI) and were used without further purification. N-Hydroxysuccinimide (NHS)-activated Sepharose 4 Fast Flow 14-atom spacer arm (between matrix and activated group) was obtained from GE Healthcare (formerly Amersham Biosciences, Uppsala, Sweden). Primary amino groups of the THs were coupled directly to active ester to form an amide linkage according to Amersham Biosciences. The contamination by T3 or T4 was always assessed by RIA assays and proved to be very low, less than 1 mol of free hormone per 10^5^ or 10^4^ mol of agarose-coupled hormone, respectively. Moreover, stability of agarose-conjugated hormone in cell culture was assessed in supernatants from 24 h lasting cultures in the presence of either T3-AG or T4-AG that were depleted of cells and agarose by centrifugation, and non-detectable levels of free hormones were found.

### Hormone determinations

Hormone determinations were done as previously described [54]. Briefly, blood was collected from the tail vein using a capillary tube coated with anticoagulant and plasma was obtained by centrifugation. The plasma levels of T3 and T4 were determined using commercial RIA kits with specific antibodies (Immunotech, Praga, Czech Republic) according to the manufacturer’s instructions. The plasma TSH level was assayed using an ELISA kit (Uscn Life Science, Inc., Wuhan, Hubei, Republic of China).

### Cell viability assay

Cell viability was measured with a fluorimetric test as previously described [55]. Briefly, 5 × 10^5^ cells/ml were settled at a final volume of 0.1 ml in 96-well flat-bottom microtiter plates, and were treated or not for 24 hours with THs or agarose bound hormones as indicated in results. We used the fluorimetric resazurin reduction method (CellTiter-Blue; Promega) to evaluate the chemosensitivity of cells treated with doxorubicin or vincristine (Sigma Chemical Co., St. Louis, MI, USA). These drugs were added in a range of 0.25–10 μM or 0.005–1 μM respectively in presence of THs or TH-AG into cells grown in 96-well plates and fluorescence (560_Ex_/590_Em_) was determined using a luminometer (NovoStar microplate reader, BMG Labtech). The percentage of viable cells was calculated by using the linear least-squares regression of the standard curve. Fluorescence was determined for 6 replicates per treatment condition, and cell viability in TH-treated cells was normalized to their respective controls.

### T lymphoma cell lines

Jurkat, CUTLL1, HuT78 and OCI-Ly12 were obtained as indicated previously [8]. Cells were cultured at optimal concentration (1–5 × 10^5^ cells/ml) in RPMI 1640 (Invitrogen, Gibco, Grand Island, NY) supplemented with 10% FBS (Gibco BRL), 2 mM glutamine (Gibco BRL), and antibiotics (Gibco BRL). The tumor cell line EL4 (ATCC, Catalog Number TIB-39), a mouse T-cell lymphoma expressing the H-2^b^ and Thy-1.2 haplotype, as well as the CD3+ and αβ T-cell receptors, was routinely tested by flow cytometry with specific antibodies against the corresponding surface markers. These cells were cultured at an optimal concentration (1–5 × 10^5^ cells/ml) in RPMI-1640 medium supplemented with 10% v/v fetal bovine serum, 2 mmol/l glutamine, and 100 mg/ml of streptomycin (all from Life Technologies). We conducted monthly tests for Mycoplasma sp. and other contaminants and quarterly cell identification by single-nucleotide polymorphism. Cells were treated at different times with THs or agarose-bound hormones (prepared as indicated above) at physiological concentration (T_3_ = 1 nM; T_4_ = 100 nM, Sigma Chemical Co., St. Louis, MI). Agarose-bound hormones were used at the same concentrations than THs. Before treatments, cells were synchronized by serum starvation as previously indicated [8]. It is worth to note that cultures incubated with physiological concentrations of THs have free hormone concentrations consistent with normal physiological levels, as previously described [56–58].

### Lymphoma model

C57Bl/6J mice were render hypothyroid in accordance with the protocol of Sterle *et al.* [9] by daily treatment with 0.5 mg/ml propylthiouracil (Sigma–Aldrich) in drinking water for 15 days. Euthyroid (with physiologic levels of circulating THs) or hypothyroid C57BL/6J animals were injected subcutaneously with syngeneic 3 × 10^5^ EL4 cells in 200 ml PBS to generate a solid tumor. After cell inoculation, hormonal treatments were maintained until the end of the experiments.

Once tumor reached a volume of 2 mm^3^, mice were randomized in two groups (*n* = 5 per group) control and Doxo treatment. Doxorubicin (2 mg/kg) was administered by intraperitoneal injection every day for 5 days. Tumor length and width were measured daily using calipers and volume was calculated by the equation V= (π/6) × length × width^2^. At the 14th day post inoculation, mice were sacrificed and tumors were excised. The animal experiments were approved by the Ethics Committee for the Use and Care of Laboratory Animals from BIOMED.

### Transient transfections with siRNA

1 × 10^7^ TCL cells were mixed with siRNA against ITGAV plus ITGB3, Stat1, CYP3A4 or a noncoding sequence (L-004565-00, L-004124-00, L-003543-00, L-008169-01, and D-001810-10, respectively, ON-TARGETplus SMART pool, THERMO SCIENTIFIC) at a final concentration of 50 nM, and electroporated at 250V for 15ms in a BTX ECM 830 electroporator. After transfection, cells were plated in RPMI 1640 medium supplemented with 10% SFB for 36 hours. The treatments corresponding to each test were then performed.

### Reverse transcription (RT) and quantitative (q)PCR

Reverse transcription and qPCR was carried out as described previously [8]. Briefly, cells line samples were homogenized in Tri-Reagent (Genbiotech SRL) and total RNA was isolated following the manufacturer’s instructions. For the *in vivo* experiment, after the animals were killed, liver and solid tumors were removed and immediately homogenized in Tri-Reagent to isolate the RNA, according to the manufacturer’s instructions. cDNA was synthesized by retrotranscription using the Omniscript kit (Qiagen GMDH). cDNA amounts present in each sample were determined using a commercial master mix for Real-Time PCR containing SYBR Green fluorescent dye (Biodynamics SRL, Buenos Aires, Argentina). qPCR reactions were carried out in an Applied Biosystems 7500. Primers sequences (Biodynamics SRL, Buenos Aires, Argentina), were designed using Primer Express software version 3.0 (Applied Biosystems, California, USA). Quantification of the target gene expression was done using the comparative cycle threshold (Ct) method according to the manufacturer’s instructions (Applied Biosystems, California, USA). An average Ct was obtained from the triplicate reactions and normalized to β2-microglobulin and then DDCt was calculated.

Human primers Sequence (5′-3′): cyp3A4 fw cattcctcatcccaattcttgaagt; cyp3A4 rv ccactcggtgcttttgtg tatct; cyp3A5 fw gctcgcagcccagtcaata; cyp3a5 rv aggtggtgc cttattgggc; cyp3A7 fw aagggctattggacgtttgaca; cyp3A7 rv atcccactggcccgaaag; cyp3A43 fw aatacgaacattgctatctc cagct; cyp3A43 rv gcttctcaccaacatatctccacat; ITGAV fw aagtgccatagctccattgggaga; ITGAV rv tcgaggatttgagatg gcaccgaa; ITGB3 fw ttcaatgccacctgcctcaacaac; ITGB3 rv acgcaccttggcctcgatactaaa; TRa fw ctgatccacattgccacaga; TRa rv ttccaggtccaccttgtctc; B2M fw agatgagtatgcct gccgtgtgaa; B2M rv tgctgcttacatgtctcgatccca.

Murine primers Sequence (5′-3′): cyp1a1 fw ataaggtcatcacgattgttttgg; cyp1a1 rv ggtacatgaggctccac gagat; cyp1a2 fw cgtcagcaagcttcagaagg; cyp1a2 rv cgatgttcagcatctcctcg; cyp2b10 fw caggtgatcggctcacacc; cyp2b10 rv caggtgatcggctcacacc; cyp3a11 fw tcacacacaca gttgtagggagaa; cyp3a11 rv gtccatccctgcttgtttgtc; B2M fw gctatccagaaaacccctcaa; B2M rv catgtctcgatcccagtagacggt.

### Immunoblotting

Samples of Jurkat cells lysates were prepared as previously described [8]. Equal amounts of proteins (50 μg) were separated by SDS–PAGE on 10% polyacrylamide gels and transferred to nitrocellulose membranes. Nonspecific binding sites were blocked with blocking buffer (5% nonfat dried milk in PBS 0.1% Tween 20). Membranes were incubated overnight at 4° C with a primary antibody CYP3A4, pStat1-Y701, Stat1, GAPDH and β-tubulin (Santa Cruz) pNF-κB-S536 (Cell Signaling). Secondary mouse or rabbit HRP-conjugated antibody (Santa Cruz Biotechnology) were incubated for 1h at room temperature. AmershamTM ECL™ Prime Western blotting detection reagent (GE Healthcare) was used to develop the protein blot. Blots were developed using ImageQuant LAS 4000 (GE Health Care). Densitometry analysis was performed by Image J (version 5.1, Silk Scientific Corporation, NIH, Bethesda, MA) software. Experimental values were referred to those obtained with the corresponding loading protein band.

### CYP3A4 activity assay

CYP3A4 activity assay was performed using the commercial kit P450 Glo (Promega) according to the manufacturer´s protocol. Briefly, cells were cultured in presence or absence of THs and inhibitors for 48 hours. Then the corresponding Luciferin and the Luminiscence detection reagent were added for the indicated time and luminescence was measured using a luminometer (NovoStar microplate reader, BMG Labtech).

### Confocal microscopy in cell cultures

Confocal microscopy analysis was performed as previously described [8]. Briefly, prior to incubation with the primary antibody (T701-Stat1, sc5808, Santa Cruz Biotechnology) cells were first fixed in cold methanol for at least 30 minutes, permeabilized with 0.1% Triton-PBS for 20 minutes and incubated overnigth at 4° C. Then cells were washed and incubated for 30 minutes with the secondary antibody, anti-rabbit-FITC (ab6717, Abcam). Nucleus compartment was defined by Propidium Iodide staining for 1 minute (0,02 mg/ml). Cells were analyzed with a Nikon Eclipse E800 confocal laser microscopy system.

## Abbreviations

THs: Thyroid hormones
T3: 3,3′,5-triiodo-L-thyronine
T4: L-thyroxine
TCL: T-cell lymphomas
CYP: Cytochrome
mTR: membrane Thyroid Hormones Receptors
Doxo: doxorubicin
VCR: vincristin
PTCL: peripheral T-cell lymphoma
TH-AG: THs-coupled to agarose
Eu: euthyroid
Hypo: hypothyroid
PTU: Propil Thiouracil
